# Risk factors associated with tick infestations on equids in Khyber Pakhtunkhwa, Pakistan, with notes on *Rickettsia massiliae* detection

**DOI:** 10.1186/s13071-021-04836-w

**Published:** 2021-07-13

**Authors:** Abid Ali, Hafsa Zahid, Ismail Zeb, Muhammad Tufail, Sulaiman Khan, Muhammad Haroon, Muhammad Tufail, Muhammad Bilal, Majid Hussain, Abdulaziz S. Alouffi, Sebastián Muñoz-Leal, Marcelo B. Labruna

**Affiliations:** 1 Department of Zoology, Abdul Wali Khan University Mardan, Khyber Pakhtunkhwa, Pakistan; 2 King Abdulaziz City for Science and Technology, Riyadh, Saudi Arabia; 3 Departamento de Ciencia Animal, Facultad de Ciencias Veterinarias, Universidad de Concepción, Av. Vicente Méndez 595, casilla 537, Chillán, Ñuble Chile; 4 Department of Preventive Veterinary Medicine and Animal Health, Faculty of Veterinary Medicine, University of São Paulo, São Paulo, Brazil

**Keywords:** Equids, Pakistan, *Rickettsia*, Risk factors, Ticks

## Abstract

**Background:**

Studies on ticks infesting equids are lacking in various parts of the world, including Khyber Pakhtunkhwa (KP), Pakistan. The aim of this study was to investigate the diversity of ticks infesting equids, associated risk factors and rickettsial detection in ticks from equids in KP.

**Methods:**

Inspection of 404 equid hosts from November 2018 to October 2019 resulted in the collection of 550 ticks. Data on tick-associated risk factors were collected from equid owners by means of a questionnaire. After morphological identification, partial DNA sequences of the tick mitochondrial 16S rRNA gene were used for taxonomic confirmation of species. Partial sequences of the *gltA* and *ompA* genes were used for *Rickettsia* detection in ticks.

**Results:**

A total of 550 tick specimens were collected on 324 (80.2%) of the equids inspected, of which 161 were horses (50%), 145 (45%) were donkeys and 18 were mules (5%). The ticks were identified as belonging to the following five species: *Rhipicephalus microplus* (341 specimens, 62% of the total ticks), *Rh. haemaphysaloides* (126, 23%), *Rh. turanicus* (39, 7%), *Rh. sanguineus* (*s.l.*) (33, 6%) and *Hyalomma anatolicum* (11, 2%). The most prevalent tick life stage was adult females (279, 51%) followed by adult males (186, 34%) and nymphs (85, 15%). Higher tick infestations were observed on male equids (relative risk [RR] 0.7432, *P* < 0.0005) and adult equids (RR 1.268, *P* < 0.0020). Ticks were frequently attached to the axial region of horses (55, 21%), sternum of donkeys (44, 21%) and belly of mules (19, 23%) (*P* < 0.04). Temporal patterns of tick infestation in association with temperature and humidity were highly significant (*P* < 0.05). Risk factors, such as animal housing (*P* < 0.0003), living management (*P* < 0.006), grazing type (*P* < 0.01) and location in hilly areas (*P* < 0.02), significantly enhanced the chances for tick infestation. Tick species analyzed in this study were phylogenetically related to species from Afghanistan, China, South Africa and Taiwan. Partial sequences of the *gltA* and *ompA* genes obtained from *Rh. microplus* and *Rh. haemaphysaloides* were 100% identical to the spotted fever group pathogen *Rickettsia massiliae*.

**Conclusions:**

Equids exposed to significant risk factors were infected by one or more of at least five tick species in KP, Pakistan, and some of the ticks harbored the human pathogen *R. massiliae*.

**Graphical abstract:**

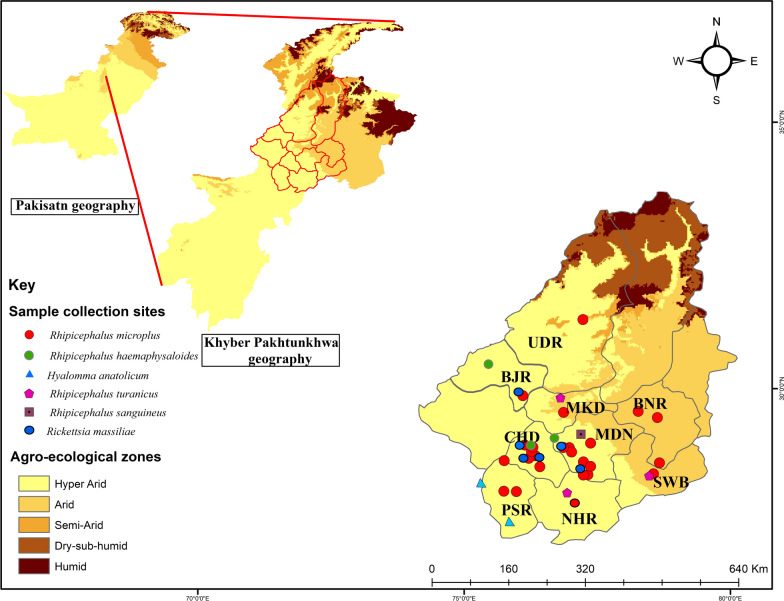

## Background

Ticks cause substantial economic losses in various communities around the world, but especially in low-income livestock holders in tropical and subtropical regions, where approximately 80% of the world’s cattle population is at risk of infestation [1]. These hematophagous ectoparasites play a major role in the transmission of pathogens, including bacteria, such as intracellular coccobacilli of the genus *Rickettsia*, and several protozoans and viruses that cause disease and are a threat to human and veterinary health [2–4]. Only 10% of tick species have been identified as carriers of pathogens; the remaining species require further research to determine whether they are vectors of disease.

Both the persistence of various life stages of ticks and their distribution depend mainly on such factors as habitat, climatic conditions, host availability and anthropogenic activities. Both biotic (vegetation structure, host availability and management) and abiotic (rainfall regimen, temperature range) factors are most likely to influence tick distributions on a global scale [5–7]. The potential impacts of climate change and transboundary movements of humans and animals on the distribution of ticks have been widely studied [8, 9]. Understanding the niche of a tick species is a complex exercise due to the multiple variables operating on the different stages of the tick life-cycle, including regulation of the patterns of tick and host abundance and their spatial encounters [10]. Some tick species exhibit ecological flexibility and adapt easily to changing climate and novel habitats [10, 11]. Efforts have been made at global, regional and local scales to characterize the major environmental factors influencing tick distributions using statistical models and correlative approaches [12]. The documentation of risk factors associated with tick distribution contributes to the design of cost-effective control measures.

Livestock activities are an integral part of Pakistan’s economy and the backbone of rural income, as more than 70% of the population live in rural areas [13]. According to the Agricultural Census Organization, the total equid population in Pakistan was 4.8 million in 2006, which rose to 5.5 million as per the census report in 2012–2013. The Pakistan Economic Survey 2018–2019 reported that the horse population was 0.4 million, donkey population was 5.4 million and mule population was 0.2 million (https://www.thenews.com.pk/print/482965-donkeys-population-increased). Khyber Pakhtunkhwa (KP) is a mountainous region of Pakistan that lacks sufficient access to transport infrastructure. Consequently, equids play important roles in providing transportation, but also in farming, pumping water and milling activities in elevated areas. Equids are also used in fairs and games, such as horse races and polo festivals. However, despite their economic importance, the welfare of equids has received far less attention than that given to other animals in Pakistan.

Equids are frequently employed in outdoor activities and are therefore exposed to tick infestations [14, 15]. Previous studies conducted in Pakistan were mostly confined to domestic animals but excluded equids, thereby limiting our understanding of the prevalence of and associated risk factors for tick infestations on these animals [13, 16, 17]. In addition, although Pakistan’s climatic conditions are suitable for the survival of tick species that parasitize domestic animals, investigations on the frequency and distribution of ticks infesting equids are lacking, as are data on *Rickettsia* associated to equid ticks that are pathogenic to humans. The present study was designed to investigate the diversity of ticks, assess the presence of *Rickettsia* and estimate the risk factors associated with ticks infesting equids in KP.

## Methods

### Study area

**Fig. 1:**
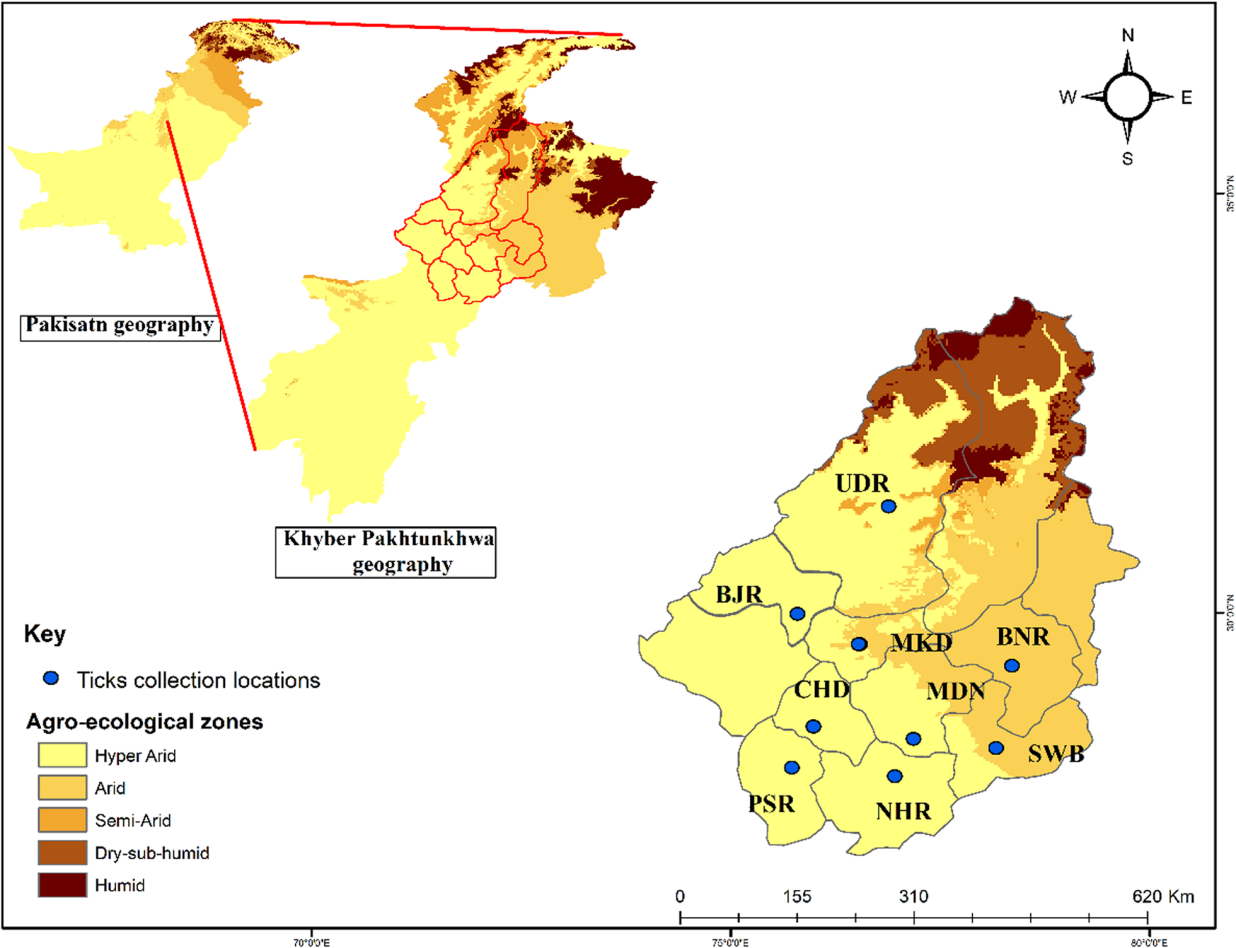
Agro-ecological zones of the selected districts of the Khyber Pakhtunkhwa (KP) region of Pakistan showing the locations (blue circles) at which ticks were collected. The study districts were: Bajaur (*BJR*), Upper Dir (*UDR*), Malakand (*MKD*), Charsadda (*CHD*), Peshawar (*PSR*), Mardan (*MDN*), Nowshera (*NHR*), Buner (*BNR*), Swabi (*SWB*)

We selected nine districts in northern, southern and central KP (northwestern geographic state of Pakistan, previously known as the North-West Frontier Province) as study areas: Charsadda (71.669079°E, 34.209086°N), Mardan (72.079179°E, 34.161767°N), Nowshera (72.005571°E, 33.988264°N), Peshawar (72.378867°E, 34.093417°N), Buner (72.484021°E, 34.450940°N), Malakand (71.863614°E, 34.519290°N), Upper Dir (71.995056°E, 35.066089°N), Swabi (72.394640°E, 34.119706°N) and Bajaur (71.621760°E, 34.634959°N). Based on access, nine regions of each of these districts were selected for tick sampling. A global positioning system (GPS) was used to obtain the geographic coordinates data and loaded onto a Microsoft Excel (Microsoft Corp., Redmond, WA, USA) spreadsheet to develop a distribution map for the study area using ArcGIS 10.3.1 (Fig. 1).

### Ethical consent and information collection

Ethical consent was obtained from the advanced studies and research board of the Abdul Wali Khan University Mardan. Oral/written consent was obtained from the owner of equids for the collection of ticks. A questionnaire was provided to equid holders that assisted in collection of data such as: host-related information (gender and age), eco-geography and herd management.

### Tick collection and morphological identification

Ticks were collected between November 2018 and October 2019 from horses, donkeys and mules at different sites (villages, towns) in tehsils (i. e. administrative divisions) within the nine districts (Fig. 1). Two visits per month were made to each area, and information related to climate was retrieved. In infested areas, ticks of all stages were removed from the base of the tail, ears, perianal area, sternum, scrotal and belly, using forceps without damaging the morphological features of the ticks. The collected ticks were placed in vials containing 100% ethanol and brought to the laboratory for identification.

Collected ticks were identified morphologically under a StereoZoom microscope (HT StereoZoom) and following taxonomic keys [18, 19]. The specimens were preserved in 100% ethanol for later molecular analysis.

### DNA extraction and PCR

Tick specimens were rinsed in distilled water and 70% ethanol and dried prior to DNA extraction [17]. Specimens were cut into pieces with a sterilized scalpel inside Eppendorf tubes. Genomic DNA of 91 randomly selected ticks was extracted using the phenol–chloroform method [20, 21]. Sterile conditions were maintained throughout the process. The concentration of DNA in each sample was quantified using a spectrometer (Thermo Fisher Scientific, Waltham, MA, USA). The extracted DNA was stored at − 20 °C.

Conventional PCR targeting a fragment (approx. 460 bp) of the tick mitochondrial 16S rRNA gene was carried out as previously described [22] to verify successful extractions (Table 1). A real-time PCR (StepOne; Applied Biosystems, Thermo Fisher Scientific, Waltham, MA USA) was performed to detect a short fragment (147 bp) of the *Rickettsia* citrate synthase-encoding gene (*gltA*) using primers CS-5 and CS-6 and an internal fluorogenic probe (6-carboxyfluorescein [6-FAM]) [2]. Positive samples were subjected to two conventional PCR assays, one to obtain a larger sequence (401 bp) of the rickettsial *gltA* gene, using primers CS-78 and CS-323 [2], and the other to amplify a 532-bp fragment of the rickettsial outer membrane protein A (*ompA*) gene [23]. Conventional PCR reactions consisted of a 25-µl mix (2 µl genomic DNA, 1 µl of each primer, 8.5 µl PCR water and 12.5 µl master mix; Thermo Fisher Scientific). PCR cycling parameters were as previously reported [2, 22, 24] (see Table 1). PCR products were electrophoresed in 1.5% agarose gels, and the results were captured with GelDoc (UVP BioDoc-It imaging system; Analytik Jena AG, Jena, Germany). Amplicons of the expected size were purified with ExoSAP-IT (Thermo Fisher Scientific) and sequenced using the BigDye Terminator v3.1 Cycle Sequencing Kit (Applied Biosystems, Thermo Fisher Scientific) in an ABI 3500 Genetic Analyzer automated device (Applied Biosystems, Thermo Fisher Scientific).

**Table 1:** List of primers used for the amplification of target genes of ticks and *Rickettsia* spp.

Organism/gene	Primer	Primers sequences (5′–3′)	Product size (bp)	References
Tick 16S rRNA	16S + 1	CCGGTCTGAACTCAGATCAAGT	460	[22]
	16S − 1	GCTCAATGATTTTTTAAATTGCTG		
*Rickettsia* spp./*gltA*	CS-5^a^	GAGAGAAAATTATATCCAAATGTTGAT	147	[2]
	CS-6^a^	AGGGTCTTCGTGCATTTCTT		
*Rickettsia* spp./*gltA*	CS-78	GCAAGTATCGGTGAGGATGTAAT	401	[2]
	CS-323	GCTTCCTAAAATTCAATAAATCAGGAT		
*Rickettsia* spp./*ompA*	Rr190.70	ATGGCGAATATTTCTCCAAAA	532	[24]
	Rr190.701	GTTCCGTTAATGGCAGCATCT		

*gltA* Rickettsial citrate synthase-encoding gene, *ompA* rickettsial outer membrane protein A

^a^These primers were used in a real-time PCR assay with the following internal probe: 5′ 6-FAM CAT TGT GCC ATC CAG CCT ACG GT- BHQ-1 3′

### Sequence and phylogenetic analyses

The obtained sequences were assembled and trimmed in SeqMan v 5.00 (DNASTAR, Madison, WI, USA) and subjected to BLASTn analyses (www.ncbi.nlm.nih.gov/blast) to infer closest identities with the organisms available in GenBank [25]. Alignments with our sequences and GenBank-retrieved sequences were constructed with CLUSTAL W in BioEdit [26, 27] manually edited with GeneDoc [28]. A phylogenetic tree for the tick mitochondrial 16S rRNA gene was implemented in MEGA X [29] and constructed using the neighbor-joining method with 1000 bootstrap replicons [30]. For alignment of the *ompA* rickettsial gene, we constructed a phylogenetic tree in MrBayes using Bayesian statistics [31], with four independent Markov chain runs for 1,000,000 metropolis-coupled Markov chain Monte Carlo (MCMC) generations, sampling a tree every 100th generation. The first 25% of the trees represented burn-in, and the remaining trees were used to calculate the Bayesian posterior probability.

### Statistical analysis

The recorded observations were assembled and arranged in the spreadsheets of Microsoft Excel V 2016. The chi-square test (χ^2^) was used for differences, and relative risk (RR) was analyzed using GraphPad Prism v. 5.00 (GraphPad Software Inc., San Diego, CA, USA), with the 95% confidence interval (CI). Significance was set at *P* < 0.05.

## Results

### Demographic characteristics of the study population

A total of 404 animals were sampled, including 200 horses, 171 donkeys and 33 mules, of which 324 (80.2%) were found to be infested with ticks (mean infestation 110.3, standard deviation [SD] 80.79, 95% CI − 90.35–311). Of the equids inspected, horses were the most infested (161, 85.5%) followed by donkeys (145, 80%) and mules (18, 55%). The highest tick burdens were found in equids inspected in Peshawar (103, 19%), Mardan (101, 18%), Nowshera (100, 18%), Swabi (96, 12.5%) and Charsadda (65, 11%); the lowest burdens were observed in Upper Dir (11, 2%), Malakand (22, 4%), Bajaur (24, 4%) and Buner (27, 5%) (Fig. 1; Table 2). The geography of each collection point significantly influenced the pattern of tick infestation on the hosts (*P* < 0.002).

**Table 2:** Ticks collected from equids in selected districts of Khyber Pakhtunkhwa, Pakistan, between November 2018 and October 2019

Districts	Tick species	Total no. collected ticks	Ticks processed for molecular analyses^a^	No. of ticks yielding rickettsial DNA	Host infested with *Rickettsia*-positive ticks
Upper Dir	*Rhipicephalus. microplus*	11	4 N, 1 M, 3 F	1 N	Mule
Bajaur	*Rh. haemaphysaloides*	17	3 N, 1 M, 2 F	1 N	Horse
	*Rh. microplus*	07	2 N, 1 F	1 N	
Mardan	*Rh. haemaphysaloides*	51	2 N, 1 F	1 N	Horse
	*Rh. microplus*	8	2 N, 1 F	1 N	
	*Rh. turanicus*	9	1 N, 1 F		
	*Rh. sanguineus*	33	3 N, 1 M, 2F		
Peshawar	*Rh. microplus*	92	3 N, 3 F	2 N^b^	Donkey
	*Hyalomma anatolicum*	11	3 N, 1 M, 3 F		
Charsadda	*Rh. haemaphysaloides*	85	3 N, 2 F	1 N	Horse
	*Rh. microplus*	10	2 N, 1 M, 1 F	1 N	
Malakand	*Rh. microplus*	17	1 N, 1 F	1 N	Horse
	*Rh. turanicus*	3	2 N, 1 M, 1 F		
Swabi	*Rh. microplus*	82	2 N, 1 F	1 F	Horse
	*Rh. turanicus*	14	3 N, 1 M, 2 F		
Nowshera	*Rh. microplus*	87	2 N, 3 F	1 N	Mule
	*Rh. turanicus*	13	2 N, 1 M, 3 F		
Buner	*Rh. microplus*	27	6 N, 1 M, 5 F	1 F	Donkey
	Total	550	91 (46 N, 9 M, 36 F)	13 (14%)	

^a^PCR assays targeting the tick 16S rRNA gene and the rickettsial *gltA,* and *ompA* gene;* N* nymphs,* M* males,* F* females

^b^Both nymphs of *Rh. microplus* were from the same donkey

**Fig. 2:**
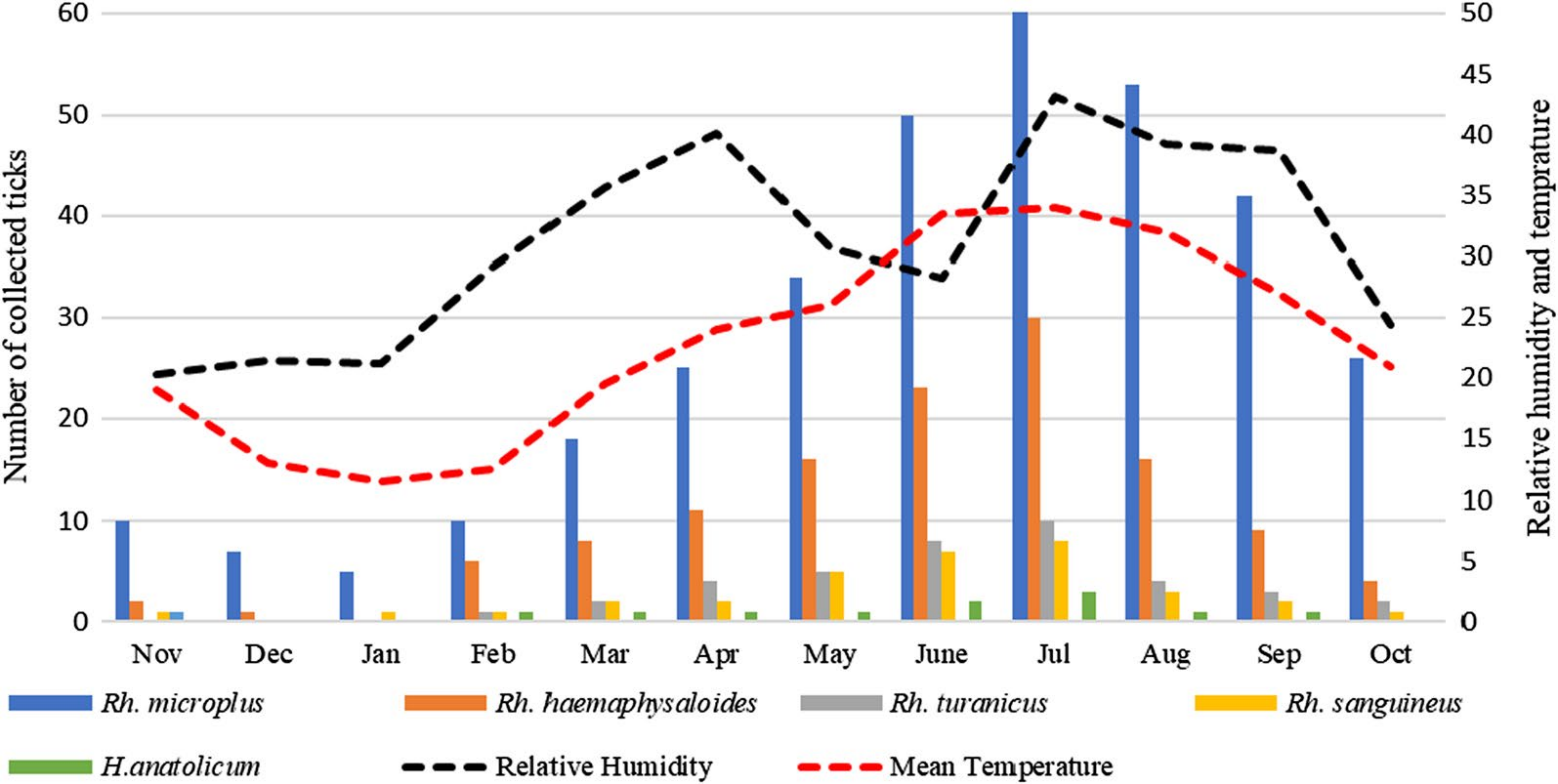
Temporal distribution of tick species collected during this study

The trend line of tick infestation increased from May (61, 11%) to August (77, 14%) of 2019, with the highest tick infestation found to be throughout the summer (*P* < 0.0354) and the lowest tick infestation during the winter between November (13, 2.3%) and January (6, 1%) (*P* = 0.1907) (Fig. 2). Linear regression analysis showed a significant association between tick infestation and high humidity (*r*^2^ = 0.9006, *P* < 0.0001) and temperature (*r*^2^ = 0.75827, *P* < 0.0002).

### Ticks and equid populations

A total of 550 collected ticks were morphologically identified into two genera and five species as follows: *Rhipicephalus* (539 specimens, 98% of all ticks) and *Hyalomma* (11, 2%)*.* The most numerous tick species was *Rhipicephalus microplus* (341 specimens, 62%) followed by *Rh. haemaphysaloides* (126, 23%), *Rh. turanicus* (39, 7%), *Rh. sanguineus* (*s.l.*) (33, 6%) and *Hyalomma anatolicum* (11, 2%).

A total of 263 ticks (48% of all collected ticks) were collected from horses, of which 185 were *Rh. microplus* (70% of the ticks from horses), 44 were *Rh. haemaphysaloides* (17%), 18 were *Rh. turanicus* (0.06%), ten were *Rh. sanguineus* (*s.l.*) (0.03%) and six were *H. anatolicum* (0.03%). From donkeys, 206 ticks (37% of all collected ticks) were collected, of which 110 were *Rh. microplus* (53% of the ticks from donkeys), 62 were *Rh. haemaphysaloides* (30%), 13 were *Rh. turanicus* (06%), 18 were *Rh. sanguineus* (*s.l.*) (8%) and three were *H. anatolicum* (1%). Mules were infested by 81 tick specimens (15% of all collected ticks), of which 46 were *Rh. microplus* (57% of the ticks from mules), 20 were *Rh. haemaphysaloides* (24%), eight were *Rh. turanicus* (10%), five were *Rh. sanguineus* (*s.l.*) (6%) and two were *H. anatolicum* (2%) (Table 3).

**Table 3:** Tick stages and species collected across Khyber Pakhtunkhwa, Pakistan, from equids

Tick species	Horse (N/M/F)	Donkey (N/M/F)	Mule (N/M/F)	Total (%)	Mean (SD)	*P* value (*χ*^2^)
*Rh. microplus*	29/65/91	16/36/58	7/14/25	341 (62)	113.7 (69.57)	0.0041 (15.32, 4)
*Rh. haemaphysaloides*	10/14/20	11/21/30	3/6/11	126 (23)	38.67 (21.3)	
*Rh. turanicus*	2/6/10	0/4/9	1/4/3	39 (7)	16.33 (10.41)	
*Rh. sanguineus*	1/3/6	4/6/8	0/2/3	33 (6)	11.00 (6.557)	
*H. anatolicum*	1/3/2	0/2/1	0/0/2	11 (2)	4.333 (1.528)	

Values in table are presented as a number unless indicated otherwise

*F* Female,* M* male,* N* nymph,* SD* standard deviation

### Tick infestation on different body parts of the equid host

Different parts of the body of the equid hosts were examined for ticks. The highest infestations were observed on the axial region of horses (63, 24%), the sternum of donkeys (44, 21%) and the belly of mules (19, 23%). The lowest tick-infested body parts were the dewlap in horses (37, 14%), the axial region in donkeys (25, 12%) and the scrotum of mules (10, 12%). Statistical analysis of infestation of the body regions of the equid hosts revealed significant differences (*χ*^2^ = 18.66, 10; *P* < 0.045) (Table 4).

**Table 4:** Number of ticks collected from different body parts of the equid hosts

Infested body parts	Horse,* n* (%)	Donkey,* n* (%)	Mule,* n* (%)	Total,* n* (%)	Mean (SD)	*P* value (χ^2^)
Dewlap	37 (14)	39 (19)	15 (19)	91 (17)	30.33 (13.32)	0.045 (18.66, 10)
Belly	39 (15)	34 (17)	19 (23)	90 (16)	30 (11.5)	
Sternum	38 (14)	44 (21)	11 (14)	93 (17)	31 (17.58)	
Udder	46 (17)	33 (16)	12 (15)	91 (17)	30.33 (17.16)	
Axial region	63 (24)	25 (12)	14 (17)	107 (19)	35.67 (23.86)	
Scrotum	40 (15)	31 (15)	10 (12)	78 (14)	26 (17.06)	
Total	263 (48)	206 (37)	81 (15)	550 (100)	183.3 (93.09)	

### Assessment of risk factors associated with tick infestations

Several independent variables (Table 5) potentially associated with tick infestations on equids were analyzed and the risk factors calculated. Analysis of infestation according to sex revealed that there were more tick infestations on male equids than on female equids, with the difference being statistically significant in the tick acquisition. When age was considered, the highest tick infestation was found on adult hosts aged 4–6 years compared to hosts aged 1–3 years. Most of the equids examined were in the plain areas, but tick infestations were predominantly found on equids in hilly areas, and the association between a high risk of tick infestation and equids in hilly areas was significant. When animal housing was considered, the tick infestation rate was found to be higher on equids in muddy systems than those maintained in concrete and semi-concrete housing. The risk of tick infestation was also higher in those managed in mud housing than in those in open grazing systems. Equids raised in a herd were found to have a higher risk of tick infestation than those reared solely. Providing fresh or stored food to the equids had no impact on the tick infestations (Table 5).

**Table 5:** Risk factors associated with tick infestation on equid hosts

Variables	Condition	No. examined equids	No of tick-infested equids (%)	Mean (SD)	Relative risk (95% confidence interval)	*P* value (*χ*^2^)
Gender	Male	330	294 (91)	312.0 (25.46)	0.7432 (0.64–0.85)	0.001 (12.05,1)
	Female	74	30 (9)	52.00 (31.11)		
Age groups (years)	1–3	100	50 (15)	75.00 (35.36)	1.268 (1.10–1.45)	0.002 (9.548, 1)
	4–6	304	274 (85)	289.0 (21.21)		
Altitude	Hilly areas	140	140 (43)	143.5 (4.950)	0.8485 (0.73–0.97)	0.02 (5.562, 1)
	Plain areas	264	184 (57)	220.5 (51.62)		
Housing	Mud	213	203 (63)	208.0 (7.071)	–	–
	Concrete	56	38 (11)	38.00 (25.46)	0.6949 (0.58–0.81)	0.001 (13.11, 1)
	Semi-concrete	135	83 (26)	174.0 (55.15)	0.8268 (0.71–0.95)	0.01 (6.645, 1)
Grazing	Open	246	229 (71)	237.5 (12.02)	0.8293 (0.72–0.94)	0.01 (7.597, 1)
	Domesticated	158	95 (29)	126.5 (44.55)		
Living management	Single	115	63 (19)	89.00 (36.77)	1.230 (1.07–1.40)	0.01 (7.921, 1)
	Herd	289	261 (81)	275.0 (19.80)		
Food supply	Fresh	295	238 (73)	266.5 (40.31)	0.9902 (0.85–1.14)	0.9 (0.0171, 1)
	Stored	109	86 (27)	97.50 (16.26)		

### Molecular confirmation of taxonomic identification of ticks

**Fig. 3:**
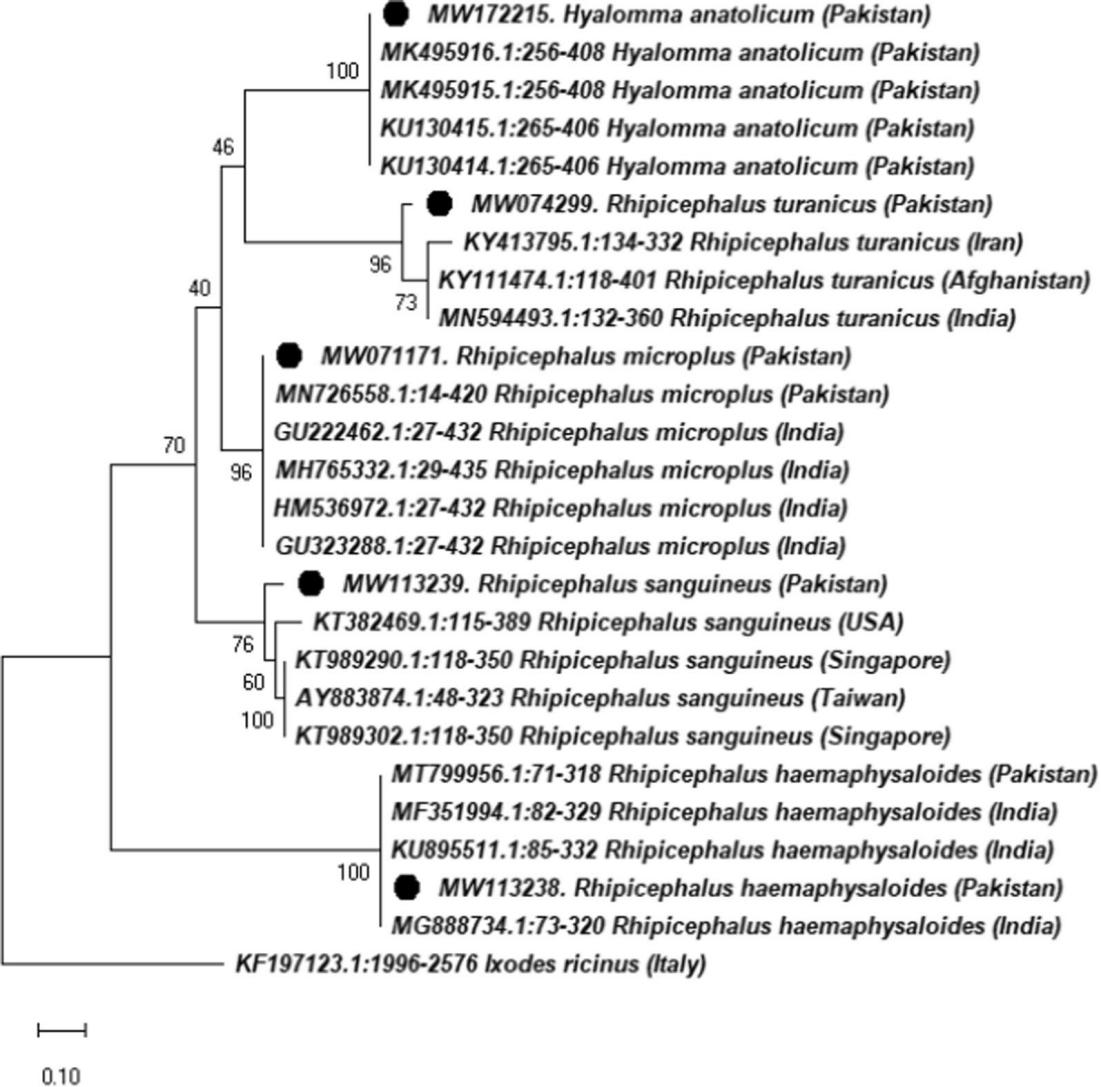
Phylogenetic analysis based on 16S rRNA gene partial sequences of *Rhipicephalus* species and *Hyalomma anatolicum* from Pakistan using neighbor-joining method in Mega X. Accession numbers are followed by species and country names. The bootstrap values are shown at each node (1000). The bar represents 0.1 substitutions per site. Sequences obtained in the present study are denoted with black circles

Partial sequences of the tick mitochondrial 16S rRNA gene were successfully obtained from *Rh. microplus* (56 specimens), *Rh. turanicus* (15 specimens), *Rh. sanguineus* (*s.l.*) (2 specimens), *Rh. haemaphysaloides* (12 specimens) and *H. anatolicum* (5 specimens). BLAST of obtained 16S rRNA sequences for *Rh. microplus*,* Rh. haemaphysaloides *and *H. anatolicum* resulted in the screening of homologous sequences from Pakistan, India and China that showed 98–99% identity. The BLAST of *Rh. turanicus* showed closest identity (95–97%) with conspecific sequences reported from India, Afghanistan, Iran, South Africa and China, and the *Rh. sanguineus* (*s.l.*) sequences showed highest identities (96–98%) with conspecific sequences from Taiwan, USA and Singapore. The resultant consensus sequences after trimming were deposited in the GenBank for each species; *Rh. microplus* (accession MW071171), *Rh. turanicus* (MW074299), *Rh. sanguineus* (*s.l.*) (MW113239), *Rh. haemaphysaloides* (MW113238) and *H. anatolicum* (MW172215). The phylogenetic analysis of the collected five species revealed an evolutionary relatedness with homologous species reported in Pakistan’s neighboring countries. In a phylogenetic tree, the obtained sequences of prevalent tick species clustered with the same species reported in the bordering countries (Fig. 3).

### Detection of rickettsial DNA in ticks

**Fig. 4:**
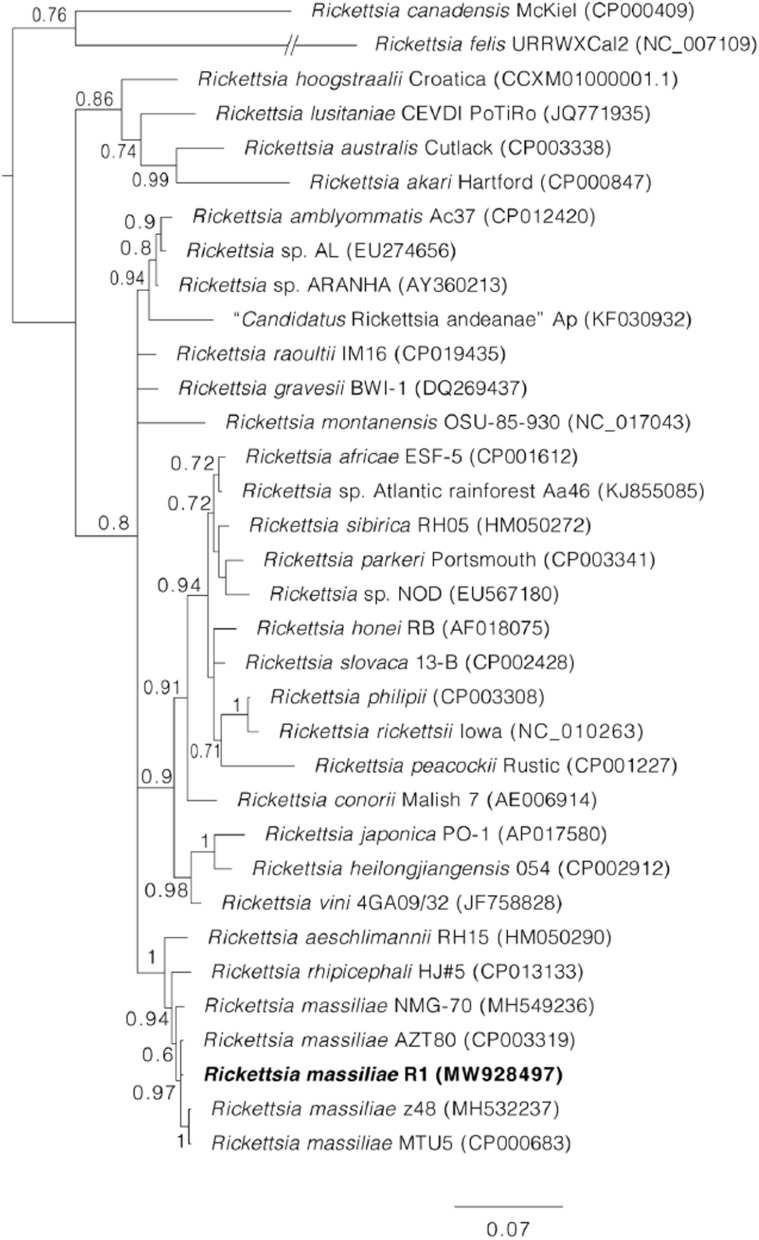
Bayesian phylogenetic tree inferred from partial sequences of the rickettsial outer membrane protein A (*ompA*) gene of *Rickettsia* spp. The tree is drawn to scale with the scale bar indicating nucleotide substitutions per site. Values of Bayesian posterior probabilities are indicated above or below each branch. The sequence of *Rickettsia massiliae* detected in ticks from Pakistan is highlighted in bold

The DNA of 91 tick specimens were molecularly tested by real-time PCR, which detected rickettsial DNA in 23 specimens of ticks (*Rh. microplus*,* Rh. turanicus*, *Rh. haemaphysaloides* and *H. anatolicum*). Among these, ten speciments showed high Cq values (> 35) and were excluded from further analyses. The remaining 13 samples were found to be positive for *Rickettsia* through conventional PCR targeting the rickettsial *gltA* and *ompA* genes. These *Rickettsia*-positive ticks included *Rh. microplus*-infected ticks (10/91, 11%) from all sampled districts, and *Rh*. *haemaphysaloides*-infected ticks (3/91, 3%) from Bajaur, Mardan and Charsadda districts. No *Rickettsia* spp. were detected in *Rh. turanicus*,* Rh. sanguineus* (*s.l.*) and *H. anatolicum* through PCR. A consensus partial sequence (333 bp) of the highly conserved *Rickettsia gltA* gene, detected in *Rh. microplus* and *Rh*. *haemaphysaloides*, was 100% identical to several sequences of *R. massiliae* from GenBank (MF002497, KY069259, KT588058, MT309038, MT309037, JN043507, CP000683, U59719). From these same ticks, a consensus partial sequence (592 bp) of the highly polymorphic *Rickettsia ompA* gene was 100% identical to a sequence of *R. massiliae* from GenBank (CP003319). The obtained sequences of *gltA* and *ompA* were uploaded to GenBank with accessions numbers MW922872 and MW928497 respectively. The obtained sequence of the *ompA* gene for *Rickettsia* spp. clustered with sequences of *R. massiliae* in a phylogenetic tree (Fig. 4)*.*

## Discussion

Ecological changes and an increasingly evident increase in global temperature have widely impacted the distribution of ticks and tick-borne diseases, ultimately increasing their threat to public and veterinary health [9, 10]. Based on spatial distribution and molecular evidence, it has been suggested that global climate change has played a significant role in the spread of tick species [10]. Previously, we reported numerous tick species infesting diverse hosts, including equids (horse), in selected districts of KP, Pakistan [13, 17]. However, a knowledge gap remained on the identity and effect of various factors affecting ticks infesting equid hosts and their associated *Rickettsia* spp. To fill this gap, we designed this study to investigate equid hosts for tick infestation, associated risk factors and the molecular phylogeny of these ticks in selected districts of KP, Pakistan. The collected ticks from five selected districts were categorized into two genera which included five medically important tick species: *Rh. microplus*, *Rh. haemaphysaloides*, *Rh. turanicus*, *Rh. sanguineus* (*s.l.*) and *H. anatolicum*. These ticks were found to be infesting equid hosts (horses, mules, donkeys) throughout the year in extreme cool to extreme hot weather conditions. The bacterium *Rickettsia massiliae* was detected in all *Rh. microplus* ticks collected in all nine study districts and in *Rh. haemaphysaloides* collected in Bajaur, Mardan and Charsadda districts. Ecological conditions and biotopes drive the geographic distribution and the risks associated with the possible harmful effects of tick species. The favorable environmental conditions in the region extend tick adaptation and also result in infestation of novel hosts [13, 16, 17].

Risk factors, such as age, altitude, housing type, grazing, living management and food conditions, of equid hosts with respect to tick infestation were statistically significant, with the exception of food conditions. A strong correlation was observed between the intensity of tick infestation and temperature and relative humidity, with the highest infestation recorded in the summer (June–August) and lowest infestation recorded in the winter (December–January); these results agree with previous reports in the region [16, 17, 32]. The variable climatic conditions of the KP favor tick survival and reproduction, resulting in high infestations on various hosts [17, 33].

The younger age group (1–years) of equid hosts was found to have lower tick infestations, possibly correlated with a strong immune response, shorter exposure to questing ticks, shorter time in free grazing conditions and frequent grooming, all of which protect younger equids from tick infestation [34–36]. Higher tick infestations on adult hosts may be due to the larger surface area of the body, which enhances the chances of tick attachment, frequent use and ultimate resistance to acaricide and low immunity [32, 36–38]. The muddy-keeping system of hosts provided favorable conditions for the survival and reproduction of ticks, as compared to concrete and semi-concrete keeping systems. Host density in herds ultimately increases the chances of tick infestation, compared to animals kept alone, due to the availability of host-questing ticks detached in the herd area [39–41]. Pastures with long grasses in the hilly areas may provide favorable conditions for the questing ticks where they await and attach to open-grazing equids.

Ticks collected across the different study regions belonged to two genera and five species. The most prevalent tick was *Rh. microplus*, which has been reported to the dominant tick species in the region [13, 16, 17]. The highest numbers of ticks were collected in the months of June to August, while the lowest numbers were collected in the coldest months of November to January, which is in accordance with the results of a previous study [17]. It is important to mention that several *Haemaphysalis* and *Hyalomma* species other than *H. anatolicum* are also abundant tick species in Pakistan [13, 16, 17], but these latter species were not found to infest the equids inspected in this study throughout the study period, possibly due to the availability of other suitable and accessible hosts for blood feeding. Several markers have been utilized to delineate the accurate identity of a tick species at the molecular level. For example, 16S rRNA, COX1, 12S rRNA and ITS are well-known genetic markers that have been implied to separate closely related tick species [17, 42–44, 51]. Since the objective of this work was only to identify tick species—and not population structure—we used 16S rRNA to identify the collected tick species and to establish their evolutionary relationship [43–46]. The phylogenetic analysis of the collected ticks showed the closest relation with the same species reported from other countries, including Afghanistan, China, South Africa and Taiwan.

Tick-borne *Rickettsia* spp. have been collected from several *Rhipicephalus* ticks infesting different hosts in various regions of the world [13, 47–51]. In Pakistan, reports are not available on ticks infesting equids associated with *Rickettsia* infection. In this study, *Rickettsia massiliae* was detected in *Rh. microplus* and *Rh. haemaphysaloides* using *gltA* and *ompA* rickettsial markers*.* There have been several reports of spotted fever due to *R. massiliae* in Europe [49]. Noteworthy, this is the first report of *R. massiliae* associated with equid ticks in Pakistan*.* Our findings highlight that human illness due to this spotted fever group of pathogens could be underreported in Pakistan, where to date tick-borne rickettsioses have not been reported.

## Conclusions

To our knowledge this is the first report of equid ticks from selected regions of KP. The risk factor analyses showed that temperature, humidity, age, keeping and grazing systems, sex and host species considerably affected the intensity of tick infestations. The phylogenetic tree of the collected ticks based on the tick mitochondrial 16S rRNA gene (2 genera and 5 species) revealed a close resemblance to tick species reported from Afghanistan, China, South Africa and Taiwan. The human pathogen *R. massiliae*, a tick-borne pathogenic spotted fever group* Rickettsia* species, was detected in *Rh. microplus* and *Rh. haemaphysaloides*. These results demonstrate that it is important to investigate the vector potential of ticks for other pathogens infesting equids in different regions of Pakistan to avoid the emergence of zoonotic infections. They will also provide valuable data for use in developing integrated control management strategies against ticks and tick-borne diseases in Pakistan.

## Data Availability

The datasets supporting the conclusions of this article are included within the article.
